# A Case of Invasive Metastatic Tonsillar Squamous Cell Carcinoma Complicated by Polymicrobial Wound Infection in a Previously Healthy 34-Year-Old Inmate

**DOI:** 10.7759/cureus.58190

**Published:** 2024-04-13

**Authors:** Kaylan Kelly, Arturo Carrion

**Affiliations:** 1 Osteopathic Medicine, Nova Southeastern University Dr. Kiran C. Patel College of Osteopathic Medicine, Davie, USA; 2 Correctional Medicine, Reception and Medical Center, Lake Butler, USA

**Keywords:** late metastatic recurrence, tonsillar squamous cell carcinoma, advanced wound care, prison health, head and neck tumors

## Abstract

This case report describes a distinctive presentation of invasive metastatic tonsillar head and neck squamous cell carcinoma (HNSCC) that recurred in a 34-year-old African American inmate, defying the expectations of conventional risk factors. This case underscores the significance of nuanced care in atypical HNSCC scenarios. The patient presented in October 2021 with bilateral lymphadenopathy and dysphagia, which led to the diagnosis of tonsillar squamous cell carcinoma. The patient’s treatment trajectory included radiation therapy with concurrent cisplatin, a subsequent radical right neck dissection, and immunotherapy. Complications, including abscess formation, neutropenic fever, and anemia, necessitated a multidisciplinary approach and admission to Reception and Medical Center Hospital. Cultures revealed a distinct neck mass that cultured positively for a variety of bacteria. The patient's condition was significantly improved by strategic interventions and meticulous daily wound care. This case prompts exploration into unknown factors contributing to HNSCC development in a seemingly low-risk individual, challenging conventional risk profiles. Treatment challenges, including radiation, surgery, and immunotherapy, underscore the need for a multifaceted approach. The central role of intense wound care in mitigating complications and improving the patient's quality of life is pivotal. The patient's tumor and infection highlight the urgency of improving prison sanitation. Enhanced hygiene and health screenings could have lessened the severity of the patient's condition, underscoring the need for comprehensive health measures in correctional facilities. Moreover, specialized wound care has the potential to improve outcomes and reduce health risks within incarcerated populations.

## Introduction

Head and neck squamous cell carcinoma (HNSCC) is a malignancy that typically occurs in individuals with identifiable risk factors such as tobacco use, alcohol consumption, occupational exposure, Epstein-Barr virus, and human papillomavirus (HPV) infection [1, 2]. Tonsillar cancer is the most common form of HNSCC and is typically associated with an underlying HPV infection [3]. This report describes an unusual case of recurrent invasive metastatic tonsillar HNSCC in a 34-year-old African American inmate with no known risk factors or medical history, emphasizing the significance of intense wound care in the management of complications associated with the disease. Encountering such a unique presentation prompts a thorough examination beyond the conventional risk factors associated with HNSCC. 

Patients with oropharyngeal cancer can display a range of symptoms, depending on where the tumor is located. The typical signs include a persistent sore throat, dysphagia, odynophagia, speech problems (dysarthria), a lump in the neck, and ear pain (otalgia). Additionally, patients may also experience changes in voice, unexplained weight loss, or hematemesis [4].

When suspicions of oropharyngeal cancer arise, it is imperative to follow a thorough investigative protocol. This includes various diagnostic procedures, such as radiography for dental assessment and bony invasion, though its usefulness is limited. Ultrasonography, often coupled with needle biopsy, is strongly recommended for patients presenting with a neck lump due to its high accuracy in nodal disease staging, particularly when conducted by experienced operators [4]. Magnetic resonance imaging (MRI) provides superior visualization of soft tissue tumor infiltration, remains unaffected by metallic dental restorations, and is considered the preferred method for primary tumor staging [4,5]. Computed tomography (CT) scanning aids in estimating tumor size, planning for tumor removal, and evaluating spread to neck lymph nodes or the lower mandible. Positron emission tomography (PET) combined with a CT scan is advised when conventional cross-sectional imaging fails to identify primary tumors and is valuable for detecting primary tumor recurrence [4]. Endoscopy and laryngoscopy, conducted under local or general anesthesia, meticulously examine suspicious sites to obtain biopsy samples. A biopsy of suspicious areas is crucial for a definitive diagnosis, with the type depending on the cancer's location. Human papillomavirus testing, utilizing polymerase chain reaction (PCR) to detect HPV DNA, is recommended by the American Society of Clinical Oncology for all newly diagnosed oropharyngeal cancers [4,5]. Ultimately, a comprehensive diagnostic approach integrating various imaging modalities, endoscopic procedures, and molecular testing ensures thorough evaluation and accurate characterization of oropharyngeal cancers and is integral to facilitating personalized treatment strategies and optimizing patient outcomes.

Significant advancements have been made in the diagnosis and treatment of oropharyngeal cancers, such as HNSCC. Biomarkers, particularly p16 for HPV-positive oropharyngeal cancer, have revolutionized diagnostics and treatment stratification, significantly improving survival rates [6, 7]. Immunotherapy, while promising, faces challenges due to immune-related adverse events and tumor immune microenvironment (TIME) complexities, requiring innovative strategies for toxicity reduction [6]. Biomaterial-based localized drug delivery systems offer a promising solution to counteract immunosuppressive TIME. Epigenetic studies highlight DNA methylation's critical role in oropharyngeal cancer progression, with non-invasive biopsy techniques enabling earlier diagnosis and risk assessment [6]. Drug repurposing and injectable biomaterials show potential for reversing resistance and reducing systemic toxicity, respectively. Interdisciplinary collaboration drives advancements, with ongoing research focusing on biomarker-guided treatment monitoring and novel biomaterials for immunotherapy delivery.

## Case presentation

In October 2021, a 34-year-old African American male sought medical attention at the Florida Department of Corrections (FDOC) for symptoms involving long-standing bilateral lymphadenopathy, new-onset weight loss, and dysphagia of unknown duration. An initial CT of the neck showed a 5.2 x 5.0 x 4.4 cm mass on the right side. Further imaging via PET scans revealed three enlarged and hypermetabolic lymph nodes in the right neck, showing activity in the tonsils. Subsequent biopsy with p40 immunochemistry staining confirmed squamous cell carcinoma suspected to originate from the tonsils. Further confirmation occurred during the patient's admission to the regional community hospital, where a comprehensive right neck suspension microlaryngoscopy, biopsy, and right tonsillectomy identified a nodular presence in the right tonsil. The investigation revealed invasive squamous cell carcinoma of the right tonsil, with likely cervical nodal metastasis.

In February 2022, the patient commenced a regimen of radiation therapy complemented by cisplatin chemosensitization, consisting of 43 sessions targeting the head and neck. Subsequent follow-ups demonstrated a significant reduction in the dimensions of the neck mass, with a near-complete resolution of hypermetabolism in several right neck lymph nodes, indicative of a robust response to therapy.

By May 2022, the patient was referred to an otolaryngologist for resection of the last active lymph node in the neck. In the ensuing months, the patient experienced a recurrence of symptoms, including neck pain, swelling, hoarseness, and dysphagia. In October 2022, the patient underwent a radical right neck dissection, although full excision was impeded by the mass's proximity to the carotid paravertebral space. Despite this, the surgical wound demonstrated commendable healing. However, an unforeseen complication emerged several months later, manifesting as an abscess in the remaining tumor tissue. The patient was treated with amoxicillin and clavulanic acid (Augmentin), followed by additional imaging revealing a significant enlargement of the tumor. Immunotherapy with pembrolizumab (Keytruda) was initiated. Despite an initial favorable tolerance, the patient experienced progressive growth of the mass, culminating in complications such as neutropenia and anemia. 

**Figure 1:**
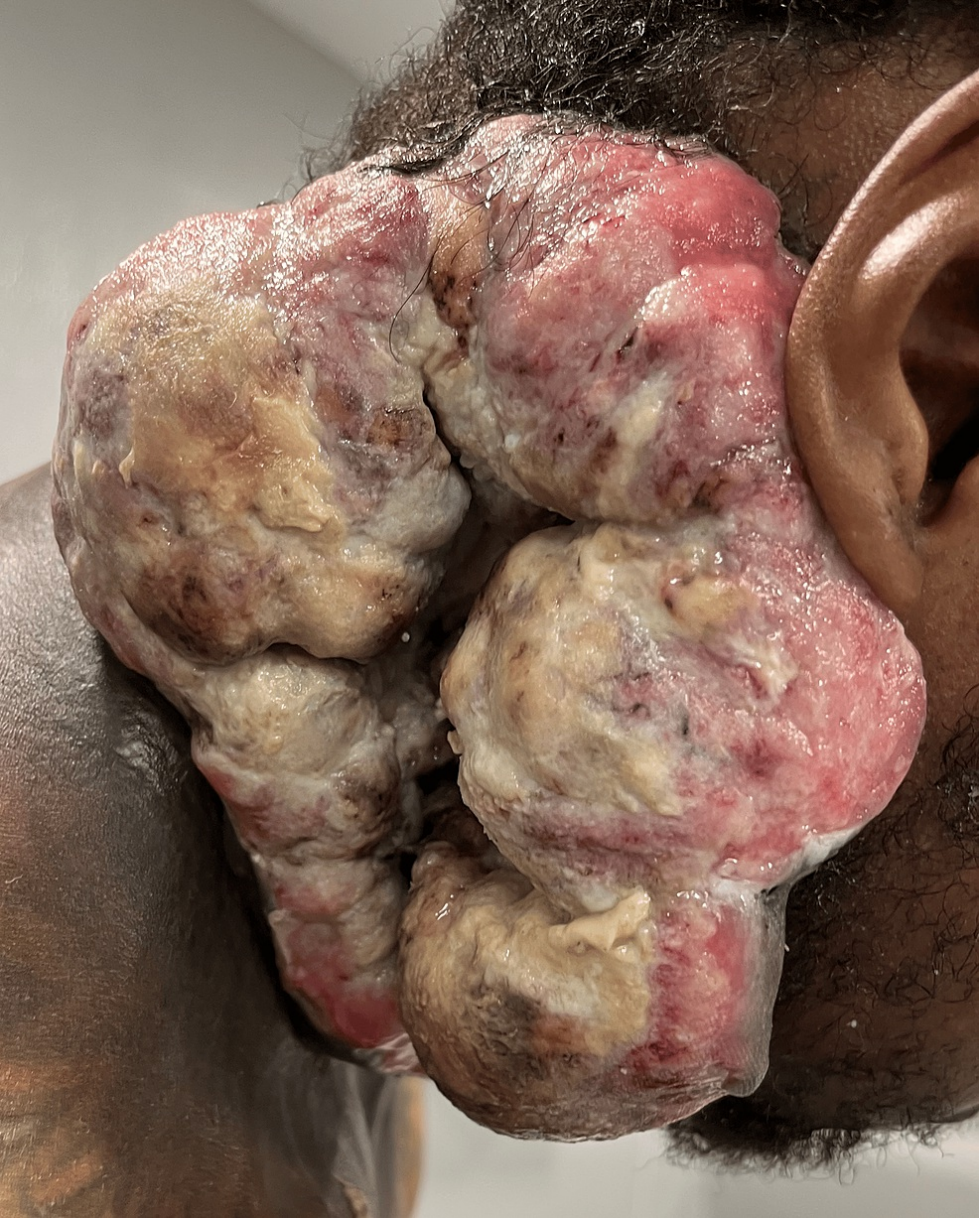
Presentation of the tumor on the admission of the patient to the hospital for neutropenic fever

In November 2023, the patient was admitted to the hospital at the Reception and Medical Center due to the onset of a fever with a white blood cell count of 1,840 cells per cubic millimeter (mm^3^) and hemoglobin (Hgb) of 6.5 g/dl. On presentation, the patient exhibited a distinct 7 x 7 cm fungating, exophytic mass with fibrinous discharge on the right side of the neck (Figure 1). Cultures from the mass confirmed the presence of *Pseudomonas aeruginosa*, group A *Streptococcus*, *Escherichia coli*, *Bacteroides vulgatus*, and *Enterococcus faecalis*. In response to this complex scenario, a range of interventions were employed. This included blood transfusions to address hemoglobin deficits, the administration of Granix to bolster neutrophil counts, and the use of appropriate intravenous antibiotic therapy to combat potential infectious agents. Concurrently, diligent daily wound care protocols were implemented to optimize healing outcomes. As a result, notable improvements were seen in the patient's clinical parameters. The patient’s fever resolved, white blood cells and hemoglobin improved, and repeat blood cultures came back negative with a reduction of suppurative exudate.

Maxillofacial specialists at the local academic hospital conducted a thorough evaluation, utilizing a CT scan to unveil the extent of the tumor's invasion into the anterior and posterior neck compartments. The findings revealed invasion into the right carotid space, involving partial encasement and displacement of the right internal and external carotid arteries, along with compression of the right internal jugular vein. Further infiltration extended to the right parotid and masticator spaces, as well as invasion at levels C3-4 and C4-5 into the right paravertebral and perivertebral soft tissues, potentially reaching into the right transverse foramen and causing possible encasement of the distal V2 segment of the right vertebral artery. The assessment also identified soft tissue edema in the hypopharynx, resulting in the narrowing of the supraglottic airway and medialization of the right vocal cord.

Considering that the tumor was classified as inoperable, the role of wound care emerged as pivotal in significantly enhancing the patient's comfort and overall well-being. This nuanced care played a crucial role in the patient's comprehensive treatment plan.

Treatment and intense wound care

**Figure 2:**
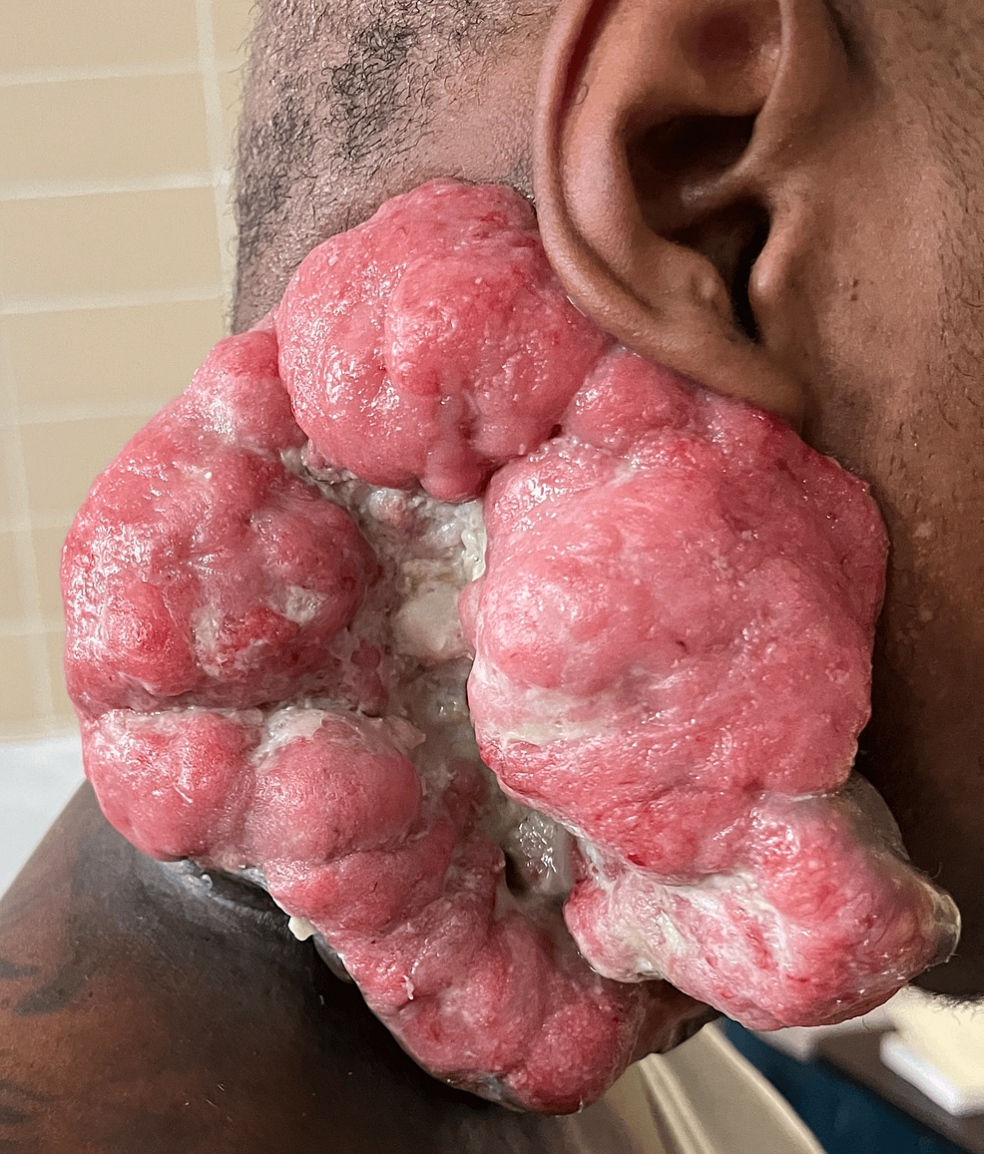
Tumor presentation after four weeks of vigorous wound care

Strategic wound care was crucial for this patient's outcome. Initial steps involved culturing the wound for pathogen identification and starting broad-spectrum antibiotics until culture results were available. Dakin's 0.50% solution was then used to cleanse the wound and prevent infections. For the unique tumor condition, Santyl (Smith+Nephew, Memphis, TN) therapy removed necrotic tissues, and Xeroform gauze fortified and covered the specific tissue, secured with 4 x 4 sterile gauze, Kerlix wrap, and coverall tape changed daily. This comprehensive approach effectively managed the polymicrobial infection, significantly improving the patient's overall condition. On admission, the patient exhibited a striking 7 x 7 cm fungating, exophytic mass with fibrinous discharge on the right side of the neck (Figure 1). Following four weeks of meticulous wound care, the fibrinous exudate resolved, revealing a pristine expanse of skin primed for regeneration (Figure 2). Prior to wound care, the patient's discomfort and embarrassment were notable, stemming from the unsettling sight, discharge, and odor coming from the wound. The intervention of a wound care specialist significantly enhanced the patient's quality of life by addressing the appearance and odor, resulting in notable improvements.

## Discussion

The significance of unknown factors in the development of invasive HNSCC in this patient raises questions about genetic, environmental, dietary, and microbiome influences. Despite the absence of traditional risk factors, the aggressive nature of the tumor suggests underlying contributors that warrant further investigation.

Genetic testing for hereditary cancer syndromes could provide valuable insight into the etiology of HNSCC without other obvious causes. The genetic syndrome most commonly associated with HNSCC is Fanconi anemia, an autosomal recessive genomic instability syndrome associated with bone marrow failure, leukemia, congenital defects, and sensitivity to cross-linking agents [8]. Additionally, rare clusters of HNSCC have also been reported in families with germ-line mutations in CDKN2A and ATR [8]. Moreover, there is a hypothesis suggesting that genetic variations in pathways like DNA repair, carcinogen metabolism, and cell cycle control could elevate the susceptibility to carcinogenesis resulting from exposure to tobacco or alcohol [8]. It is unknown whether this patient had any known family history or was genetically tested.

Assessment of environmental exposures during a patient’s life could also be beneficial in discovering the underlying causes of HNSCC. For example, exposure to cancer-causing air pollutants, polycyclic aromatic hydrocarbons, nitrosamines, aldehydes, and aromatic amines poses a risk for HNSCC [1, 9]. This is particularly the case in developing regions with increasing air pollution, such as India and China, where this patient did not reside [1].

Possible alternative explanations for the development of the patient's condition may include contributors such as dietary factors and the microbiome, which have gained recognition in recent decades [10,11]. In specific Asian-Pacific communities, the incidence of oral cavity cancer is connected to the consumption of areca nut products, particularly 'betel quid.' This term encompasses a range of customized blends that typically include areca nut (the source of carcinogens), betel leaf (derived from *Piper betle*), slaked lime, and/or tobacco, along with spices in accordance with local customs [1,2]. Interestingly, an investigation into the oral microbiome in HNSCC revealed a correlation with increased levels of the Lachnospiraceae and Eikenella families of microflora [12]. These families have previously been linked to periodontitis, suggesting that maintaining a healthy oral microbiome may play a role in preventing HNSCC [12]. Additional risk factors for developing HNSCC involve aging, maintaining poor oral hygiene, and having diets deficient in vegetables [1]. Although there were likely suboptimal oral hygiene and dietary deficiencies during the patient’s incarceration, it remains uncertain whether these conditions existed prior to confinement. The impact of electronic cigarettes on the risk of HNSCC is still uncertain and will become more apparent in the future. 

The emergence of the patient's tumor alongside the infection emphasizes the critical need to address unsanitary conditions within correctional facilities. Prolonged exposure to unhygienic surroundings and close contact with numerous individuals may have significantly impacted the inmate's health, potentially contributing to the development of the tumor. Improved sanitary practices could have mitigated the severity of the patient's condition. Additionally, implementing more rigorous health screenings among the incarcerated population could have led to earlier detection and intervention, potentially altering the disease's progression. Therefore, a thorough investigation into environmental factors within prisons and the development of comprehensive health screenings are essential to understanding their influence on tumor onset and progression.

Finally, it is essential to highlight the positive impact of meticulous wound care on this patient. While the prognosis for this patient appears grim, the wound care management employed could have substantial implications for future practices, especially within incarcerated communities. The visible improvement in the patient's condition serves as evidence of the effectiveness of dedicated wound management. Given the high risk of dangerous pathogens and microbes in prison settings, having specialized wound care professionals, like the one involved in this patient's treatment, holds the promise of improving health outcomes across the incarcerated population. By addressing and treating wounds with precision, these specialists not only contribute to individual patient well-being but also play a crucial role in minimizing the broader health risks associated with the confined environment of prisons.

## Conclusions

In summary, the detailed case report of a 34-year-old African American inmate grappling with recurrent invasive metastatic tonsillar HNSCC unveils critical insights into the intersection of healthcare and incarceration. Despite lacking traditional risk factors, the aggressive nature of the tumor prompts a deeper exploration into underlying factors that may have catalyzed its development. The concomitant emergence of the tumor alongside infection serves as a stark reminder of the dire need to address unsanitary conditions rampant within correctional facilities. This case underscores the urgency of implementing rigorous health screenings and bolstering sanitary practices to mitigate the adverse impact of such environments on inmate health.

Moreover, the remarkable efficacy of intensive wound care in improving the patient's condition stands as a testament to its indispensable role in correctional healthcare. The visible improvements observed underscore the potential for specialized wound management practices to revolutionize inmate healthcare, promising enhanced well-being and reduced health risks within prison settings. By utilizing comprehensive diagnostic approaches and prioritizing meticulous wound care, this case report advocates for a shift in correctional healthcare, one that prioritizes inmate health and fosters better outcomes through targeted interventions.

This case sheds light on the complex connection between health and incarceration, urging prompt action. It calls upon healthcare providers and correctional authorities to implement reforms aimed at safeguarding the health and dignity of incarcerated individuals. By prioritizing evidence-based practices and inmate welfare, we can work towards a more equitable and compassionate correctional healthcare system, promoting justice, compassion, and public health for all.
